# Effects of acupuncture on rates of ovulation and pregnancy in women with unruptured follicular luteinization syndrome: A protocol for systematic review and meta-analysis

**DOI:** 10.1097/MD.0000000000017294

**Published:** 2019-10-18

**Authors:** Ting Xi, Yanfeng Liu, Xiaoxia Bao, Shuqi Mi

**Affiliations:** Dongzhimen Hospital Affiliated to Beijing University of Chinese Medicine, Beijing, China.

**Keywords:** acupuncture, protocol, the rates of ovulation and pregnancy, women with unruptured follicular luteinization syndrome

## Abstract

**Background:**

As a fabulous part of Oriental Medcine, acupuncture and moxibustion possesses the advantage of high safety, convenience and less adverse effects. Unruptured follicular luteinization syndrome is a common cause of infertility in women of reproductive age, which seriously affects the physical and mental health of patients. Certain studies have reported that acupuncture can improve the rate of pregnancy in women with unruptured follicular luteinization syndrome. In this protocol, the effects of acupuncture on rates of ovulation and pregnancy among women with unruptured follicular luteinization syndrome will be further explored.

**Methods:**

Electronic bibliographic databases such as: MEDLINE, EMBASE, PsycINFO, Global Health, The Cochrane Library (Cochrane Database of Systematic Reviews, Cochrane Central Register of Controlled Trials (CENTRAL), Cochrane Methodology Register), Health Technology Assessment Database, and Web of Science (Science and Social Science Citation Index), PubMed, Chinese Biomedical Databaseare, Chinese VIP Information, Chinese National Knowledge Infrastructure (CNKI), all helpful to identify relevant randomized controlled trials (RCTs) of effects of acupuncture on rates of ovulation and pregnancy among women with unruptured follicular luteinization syndrome. The pooled odds ratio of achieving a clinical pregnancy, ongoing pregnancy, or live birth were used as the main outcome and the secondary outcome includes the changes of ovarian artery dynamics before and after treatment, so as to the adverse reactions of treatment. We will use RevMan 5.3 software to help us to analyze all data and use the Cochrane evaluation manual 5.1.0 to help us to assess the methodological quality for incorporated RCTs.

**Result:**

This systematic review will provide evidence for assessing the effects of acupuncture on rates of ovulation and pregnancy in women with unruptured follicular luteinization syndrome.

**Conclusion:**

The results of this study will be a useful reference for clinical treatment with acupuncture to improve rates of ovulation and pregnancy among women with unruptured follicular luteinization syndrome.

## 1 Introduction

### 1.1 Description of the condition

Luteinized unruptured follicular follicle syndrome (LUFS) is a syndrome of failed ovulation in which the follicle does not rupture, despite of the secondary ovulatory changes, such as peak luteinizing hormone (LH), rising progesterone, or endometrial secretion transformation.^[1,2]^ The diagnosis of LUFS still needs to be carried out according to continuous monitoring of follicles. The common methods include: Laparoscopy fails to detect ovulation; Histological examination shows follicular luteinization.

LUFS is a special type of an ovulatory menstruation and a common cause of infertility. In the natural menstrual cycle, the incidence of LUFS is 11% to 23% ^[3]^ and in ovulation cycle is higher; besides, combined with endometriosis, the incidence of LUFS is 13% to 73%.^[4]^ A survey in China has showed that the incidence of LUFS was 20.2% in 80 infertile women between the age of 28 to 43. Meanwhile, the incidence of LUFS has been increasing in recent years and the recurrent rate of LUFS is much higher ranging from 78.6% to 90%.^[2]^ In addition, LUFS not only affects ovulation, but also impairs the receptivity of endometrium and interferes with embryo implantation.^[5,6]^ Lastly, LUFS causes great psychological pressure on women and seriously affects the quality of life. Therefore, it is vital to improve rates of ovulation and pregnancy in women with unruptured follicular luteinization syndrome.

### 1.2 Description of the intervention

Acupuncture is an important part of Oriental Medicine. It is a safe, convenient and almost no side effect treatment by the conduction of meridians and acupoints and the application of certain method of operation. Acupuncture includes simple acupuncture, electroacupuncture therapy, moxibustion therapy, acupoint embedding, and auricular therapy, and so on. According to the literature reports, acupuncture can improve the rate of embryo transferring,^[7]^ endometrial receptivity ^[8]^ and pregnancy outcome. Recent studies have reported that acupuncture has a certain therapeutic effect on LUFS. However, the relationship between LUFS and acupuncture has not been clarified yet. Overall, there is a lack of supportive evidence on the effects of acupuncture on rates of ovulation and pregnancy in women with LUFS. Therefore, a systematic review and meta-analysis is necessary to explore the improvement effects.

## 2 Methods and analysis

### 2.1 Study registration

The protocol has been registered on the International Prospective Register of Systematic Reviews (PROSPERO) (registration number, CRD42019119791) based on the Preferred Reporting Items for Systematic Reviews and Meta-Analyses Protocols (PRISMA-P) statement guidelines.

### 2.2 Eligibility criteria

#### 2.2.1 Research type

All randomized controlled trials (RCTs) that estimated the rate of ovulation and pregnancy of acupuncture and moxibustion interventions for women with luteinized unruptured follicles syndrome will be collected (We will include randomized controlled trials to assess the beneficial effects of the treatments, and supplement with observational studies including cohort and case–control studies for the assessment of harms). There is no unified requirement on the blinding and language of the findings.

#### 2.2.2 Participant type

As to the patients in this study, they are diagnosed as Luteinizing syndrome of unruptured follicle by clinicians based on Practical gynecological endocrinology edition^[9]^ and Diagnostic criteria for gynecological diseases edition.^[10]^ Only married women meets the inclusion criteria. Patients need to be between 20 and 42 with no contraceptive, but normal sex life more than 1 year.

#### 2.2.3 Intervention measures

Intervention group will be treated with 1 or several kinds of methods of acupuncture and moxibustion, which including acupuncture, auriculotherapy, electroacupuncture, moxibustion, fire needling, warm needling, scalp acupuncture, abdominal acupuncture, dermal needle, embedding therapy, ear seed pressure, cupping and pricking blood therapy, regardless of the form, type of needle, length of needle; while control group will be treated with drugs only, such as progesterone and Chinese herbal medicine, placebo acupuncture or no treatment at all.

#### 2.2.4 Outcome measures

The primary outcome measures will be as follows: the pooled odds ratio of achieving a clinical pregnancy, ongoing pregnancy, or live birth for women in the acupuncture group compared with the control group. The secondary outcome measures will include the changes of ovarian artery dynamics before and after treatment and adverse reactions to treatment.

#### 2.2.5 Exclusion criteria

We included only trials in which acupuncture involved the insertion of needles into traditional meridian points. The needles could be inserted into tender points or the traditional meridian points, and the needles could also be combined with other methods at the same time. Studies using therapies similar to acupuncture but no needle insertion (i.e., acupressure, laser acupuncture, and electrostimulation without needles) or studies that only compared 2 types of active acupuncture were excluded.

### 2.3 Data sources

#### 2.3.1 Electronic search

Electronic bibliographic databases such as: MEDLINE, EMBASE, PsycINFO, Global Health, The Cochrane Library (Cochrane Database of Systematic Reviews, Cochrane Central Register of Controlled Trials (CENTRAL), Cochrane Methodology Register), Health Technology Assessment Database, and Web of Science (Science and Social Science Citation Index), PubMed, Chinese Biomedical Databaseare, which all are helpful to identify relevant RCTs and will be searched from inception to February 10th, 2019. Titles and/or abstracts of studies retrieved using the search strategy (We have searched the following terms as free text terms and MeSH terms (shown in italics): (acupuncture; acupuncture therapy; auriculotherapy; electroacupuncture; Medicine, Oriental Traditional; Medicine, Traditional Chinese Medicine; moxibustion), and (luteinized unruptured follicle syndrome; LUFS)). Above terms will be translated into Chinese, which can help to search relevant findings in the Chinese databases. Endnote software 8.1 will be served as the reliable tool to exclude the duplicate articles.

#### 2.3.2 Additional search

To ensure the comprehensiveness of this research, we will retrieve other potential articles in the reference list of retrieved studies. As to the articles not included in the electronic database or related papers and journal, further consultation will be needed.

### 2.4 Study selection and data extraction

**Figure 1:**
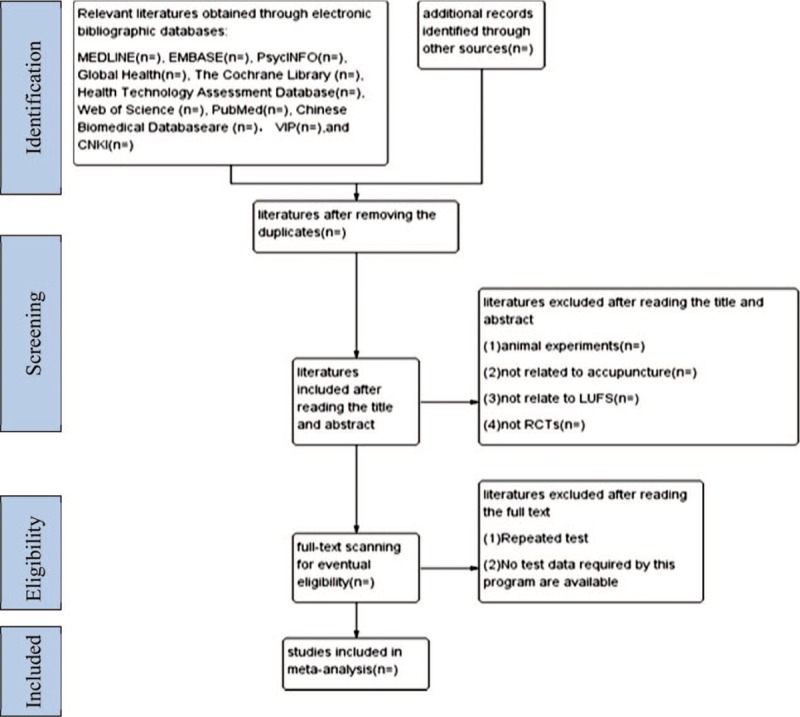
Preferred reporting itema for systematic review and meta-analysis (PRISMA) flowchart.

Two reviewers will firstly adopt Endnote software 8.1 to acquire useful findings by reading abstracts of articles obtained from databases mentioned above and to eliminate redundancies. Articles that are noticeably inapplicable based on reading the titles and abstracts will be deleted. The final list of articles will be converted to Microsoft Excel format. Then 2 reviewers will independently read through the whole findings to decide the final eligibility as a second analysis. Any disagreement between them over the eligibility of particular studies will be resolved through the discussion with a third reviewer. In order to make clear of the study selection procedure, a Preferred Reporting Items for Systematic Reviews and Meta-Analyses (PRISMA) flow chart is shown (Fig. 1).

We will extract following data: extracted information will include: age, course of disease, demographic characteristics, interventions, number of included cases, number of excluded cases, frequency of treatment, baseline characteristics, patient reported outcomes, ovulation rate, pregnancy rate, ovarian arterial blood flow dynamics index, randomized methods, allocation concealment, blind methods for subjects and personnel, data integrity of results, selective reporting of study results, and other biases; suggested mechanisms of intervention action; information for assessment of the risk of bias.

### 2.5 Data analysis

#### 2.5.1 Risk of bias in the included studies

We will use the Cochrane Handbook, version 5.1.0 to assess the quality of the included literature, which includes the random method, allocation concealment method, blind method, baseline, data integrity of the results, and selective reporting of the results. The quality of the literature was divided into grade A (low bias), grade B (medium bias), and grade C (high bias). Literature quality assessment was conducted independently by 2 researchers and cross-checked. Disagreements between the review authors over the risk of bias in particular studies will be resolved by discussion or with involvement of a third review author if necessary. Any discrepancies or unusual patterns will be checked with the study investigator. A final copy of the form from each trial will be returned to the appropriate trial investigator for verification.

#### 2.5.2 Data synthesis

The included trials will be categorized and analyzed according to the types and outcomes of acupuncture. For incomplete and missing data, the investigators will contact the author for further information. Data were summarized using RevMan 5.3 software. Continuous data were summarized using means and SDs.

The mean difference or standardized mean difference, with the 95% confidence interval, was calculated for the pooled effect size of continuous variables that were measured using the same instrument or different instruments, respectively. Homogeneity was tested by *χ*^2^ test.

#### 2.5.3 Assessment of heterogeneity

We choose *χ*^2^ test statistics to assess the heterogeneity of included studies. If *P* > .1, the fixed-effects model will be used for meta analysis. If *P* < .1 and *I*^*2*^ < 50%, heterogeneity exists among studies; however, a random effect model is used for Meta analysis within the acceptable range. If *P* < .1 and *I*^*2*^ > 50%, it indicates that there is heterogeneity among studies so that subgroup analysis and descriptive analysis will be used for in-depth discussion.

#### 2.5.4 Addressing missing data

If any data important for evaluation is missed, first we will try to contact the corresponding authors of articles through reliable ways. If the sufficient data fail to be obtained after contacting the author, we will make an analysis with the available data and discuss possible impact of missing data.

#### 2.5.5 Assessment of publication bias

Assuming that more than 10 RCTs are included, publication bias needs to be assessed by Begg funnel plot and Egger test. If an asymmetrical funnel plot or a *P* value of <.1 on Egger test, it will be supposed that publication bias exists. On the contrary, it means no publication bias if the points become symmetrically distributed on either side of the funnel plot.

#### 2.5.6 Subgroup analysis

If there exists considerable heterogeneity in the clinical researches, we will conduct a subgroup analysis based on different intervention methods (acupuncture, electroacupuncture, warm acupuncture) and different age group (20–30, 30–40, >40 years old). This will be a qualitative synthesis. Meanwhile, it is not possible to specify groups in advance due to the reason that whether the subgroup analysis will be used or not.

#### 2.5.7 Sensitivity analysis

If the heterogeneity is high, we will conduct sensitivity analyses based on the study type, sample size, methodological quality, and the effect of missing data.^[11]^

#### 2.5.8 Evidence synthesis

We will evaluate the quality of evidence of the included studies based on the guidelines of the GRADE (Grading of Recommendations, Assessment, Development, and Evaluation). The evidence quality will be ranked with 4 levels: high, moderate, low, and very low.

#### 2.5.9 Grading the quality of evidence

We will evaluate the quality of evidence for each outcome on the basis of guidelines of the GRADE (Grading of Recommendations, Assessment, Development, and Evaluation).^[12]^ The quality of evidence will be ranked with 4 levels: high, moderate, low, and very low.

## 3 Ethics and dissemination

Ethical approvals and patient consent are not applied for because the meta-analysis will be based on published research. We will submit our meta-analysis which evaluates the rates of ovulation and pregnancy in women with luteinized unruptured follicles to a peer-reviewed journal for publication or conference presentations.

## 4 Discussion

The etiology of LUFS is complex and the pathogenic factors of LUFS include endocrine disorders, drug factors, local disorders, psychological factors, and so on. It has been reported in the literature that the incidence of LUFS is related to the low level of LH and Progesterone levels in the ovulation period.^[13]^ Women with a history of endometriosis, pelvic inflammatory disease, induced labor or abortion have a higher incidence of LUFS and a higher recurrence rate. Pelvic cavity local tissue adhesion leads to the thickened surface of the ovary capsule which makes it difficult to the discharge of follicles and the formation of lutein cysts, is also known as mechanical LUFS. Long-term anxiety, nervousness and other negative emotions can increase catecholamines and PRL in the blood, which may affect ovulation. Clinical studies have found that non-steroidal anti-inflammatory drugs such as indomethacin can cause ovarian ischemia, which leads to the obstruction of mature follicles; and it may also lead to the decreased blood flow velocity during systolic ovarian systolic phase at the LH peak during ovulation which inhibiting ovulation.^[14]^ At present, the treatment of LUFS mainly takes advantage of clomiphene citrate, urinary gonadotropin (HMG), human chorionic gonadotropin (HCG), other ovulation-promoting drugs, exogenous progesterone supplementation, follicular puncture guided by ultrasonic image and IVF –ET,^[15]^ etc. However, a long-term use of hormonal drugs can damage liver and kidney function and even ovarian hyperstimulation syndrome. Follicular puncture and oviposition may cause different degrees of abdominal pain and a small amount of pelvic bleeding. All of the effects mentioned above are not satisfactory. Therefore, it is important to explore an effective, economical, less side-effect supplement and replacement therapy.

As far as we know, it will be the first attempt to perform a systematic review and meta-analysis of effects of acupuncture on rates of ovulation and pregnancy among women with LUFS. This protocol will provide referable evidence of the effects of acupuncture on rates of ovulation and pregnancy among women with LUFS. In addition, the protocol will also provide changes of ovarian artery dynamics before and after treatment and adverse reactions to the treatment. What is more, the results will provide a useful reference for clinicians to develop treatment options.

## Author contributions

**Conceptualization:** Ting Xi.

**Formal analysis:** Ting Xi, Yanfeng Liu, Xiaoxia Bao, Shuqi Mi.

**Methodology:** Ting Xi, Xiaoxia Bao, Shuqi Mi.

**Software:** Shuqi Mi.

**Writing – original draft:** Ting Xi, Shuqi Mi.

**Writing – review & editing:** Ting Xi, Yanfeng Liu, Xiaoxia Bao, Shuqi Mi.
